# Technical outcomes and postprocedural courses of mucosal incision‐assisted biopsy for possible gastric gastrointestinal stromal tumors: A series of 48 cases (with video)

**DOI:** 10.1002/deo2.264

**Published:** 2023-06-22

**Authors:** Eriko Koizumi, Osamu Goto, Shun Nakagome, Tsugumi Habu, Yumiko Ishikawa, Kumiko Kirita, Hiroto Noda, Kazutoshi Higuchi, Takeshi Onda, Teppei Akimoto, Jun Omori, Naohiko Akimoto, Katsuhiko Iwakiri

**Affiliations:** ^1^ Department of Gastroenterology Nippon Medical School Graduate School of Medicine Tokyo Japan; ^2^ Division of Endoscopy Nippon Medical School Hospital Tokyo Japan

**Keywords:** gastric subepithelial lesion, gastrointestinal stromal tumor, mucosal incision‐assisted biopsy, postprocedural course, technical outcome

## Abstract

**Objective:**

Mucosal incision‐assisted biopsy (MIAB) has been introduced as an alternative to endoscopic ultrasound‐guided fine needle aspiration for tissue sampling of subepithelial lesions. However, there have been few reports on MIAB, and the evidence is lacking, particularly in small lesions. In this case series, we investigated the technical outcomes and postprocedural influences of MIAB for gastric subepithelial lesions 10 mm or greater in size.

**Methods:**

We retrospectively reviewed cases with the intraluminal growth type of possible gastrointestinal stromal tumors, in which MIAB was performed at a single institution between October 2020 and August 2022. Technical success, adverse events, and clinical courses following the procedure were evaluated.

**Results:**

In 48 MIAB cases with a median tumor diameter of 16 mm, the success rate of tissue sampling and the diagnostic rate were 96% and 92%, respectively. Two biopsies were considered sufficient for making the definitive diagnosis. Postoperative bleeding occurred in one case (2%). In 24 cases, surgery has performed a median of two months after MIAB, and no unfavorable findings caused by MIAB were seen intraoperatively. Finally, 23 cases were histologically diagnosed as gastrointestinal stromal tumors, and no patients who underwent MIAB experienced recurrence or metastasis during a median observation period of 13 months.

**Conclusions:**

The data indicated that MIAB appears feasible, safe, and useful for histological diagnosis of gastric intraluminal growth types of possible gastrointestinal stromal tumors, even those of a small size. Postprocedural clinical effects were considered negligible.

## INTRODUCTION

Some subepithelial lesions (SELs) need to be pathologically diagnosed to differentiate gastrointestinal stromal tumors (GISTs), which are potentially malignant tumors.^1^, ^2^, ^3^, ^4^ However, it is considered difficult to obtain an adequate amount of tissue for histology by conventional endoscopic biopsy because SELs are usually covered with normal epithelium. At present, endoscopic ultrasonography‐guided fine‐needle aspiration (EUS‐FNA) is the gold standard for the tissue sampling of SELs with high diagnostic accuracy.^3^, ^4^, ^5^, ^6^ On the other hand, although improvements have been made with the introduction of new needles such as the Fork‐tip needle or Franseen needle, this technique potentially has an unignorable sampling error in gastric SELs, particularly in small lesions: a previous study reported that the probability of obtaining adequate samples in gastric SELs was 74%–96% and decreased to 68% in cases smaller than 20 mm.^7^, ^8^, ^9^, ^10^, ^11^

Mucosal incision‐assisted biopsy (MIAB), also called single‐incision needle‐knife biopsy (SINK biopsy), is an endoscopic submucosal dissection (ESD)‐associated tissue sampling technique in which the epithelium covering SELs is cut and the tissue directly sampled with biopsy forceps from the exposed tumor. Previous studies have reported that the diagnostic ability of MIAB for SELs is 82%–93%, which is comparable to that of EUS‐FNA, and 72%–93% in cases smaller than 20 mm, which is even higher than EUS‐FNA.^11^, ^12^, ^13^, ^14^, ^15^, ^16^, ^17^, ^18^, ^19^ In 2022, the European Society of Gastrointestinal Endoscopy guideline for SELs cited MIAB as an alternative method for tissue sampling of SELs to EUS‐FNA.^20^ In contrast, MIAB for SELs in Japan is still controversial due to a lack of evidence.

In this study, we investigated the short‐term technical outcomes of MIAB to evaluate the diagnostic ability and the postprocedural course to assess the clinical feasibility of this technique in possible gastric GISTs, including small lesions.

## METHODS

This was a consecutively collected retrospective case series in a single center. This study was approved by the Institutional Review Board of our institution. Written informed consent for the endoscopic procedure and retrospective use of clinical data was obtained from all patients beforehand.

### Indication of MIAB

**FIGURE 1:**
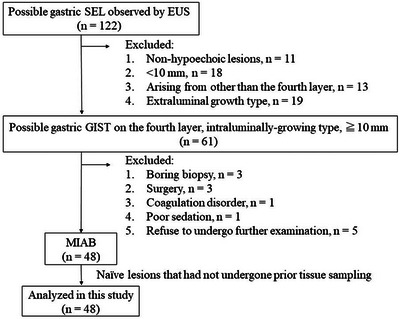
Flowchart of patients in this study: Of 61 patients with intraluminally growing gastric subepithelial lesions (SELs) larger than 10 mm that were shown as hypoechoic lesions arising from the fourth layer in endoscopic ultrasonography findings, 48 patients underwent mucosal incision‐assisted biopsy for gastric SELs. All cases of them had naïve lesions that had not undergone prior tissue sampling and were analyzed in this study.

Gastric SELs larger than 10 mm that had been noted on esophagogastroduodenoscopy or computed tomography were applied to EUS (GF‐UE260; Olympus Medical Systems Corp., Tokyo, Japan), and those that were shown as hypoechoic lesions arising from the fourth layer were considered as possible GISTs. MIAB was indicated for intraluminal growth type possible GISTs with a diameter ≥10 mm on EUS. Of 122 patients with gastric SELs who underwent EUS between October 2020 and August 2022, we retrospectively extracted 61 cases with intraluminal growth type possible GISTs with a diameter ≥10 mm. Among them, cases in which a boring biopsy was performed due to ulceration instead of MIAB were excluded. In reference to blood tests that had been performed in advance, patients showing severe anemia or coagulation disorder were excluded. In patients who had a rapidly growing lesion or who desired tumor resection directly, surgery was performed without preoperative tissue collection. In addition, cases in which further procedure should not be indicated due to poor sedation and cases in which patients refused to undergo further examination were also excluded. Finally, patients who underwent MIAB for the tissue sampling of gastric SELs were retrospectively collected and among them, those with naïve lesions that had not undergone prior tissue sampling were analyzed in this study (Figure 1).

### Mucosal incision‐assisted biopsy

The MIAB procedure was conducted by either of two endoscopists who had performed more than 100 gastric ESD procedures using a single‐channel endoscope equipped with a water‐jet function (GIF‐Q260J, H260Z; Olympus Co., Ltd.) and a transparent hood (D‐201‐11804, D‐201‐11304; Olympus). VIO300D (Erbe Elektromedizin GmbH, Tubingen, Germany) was used as an electrosurgical unit in all cases. MIAB was performed on the same day as preoperative EUS or on a separate day as an outpatient. If antithrombotic medications were taken, MIAB was performed during hospitalization upon the withdrawal of these agents according to the guidelines.^21^

**FIGURE 2:**
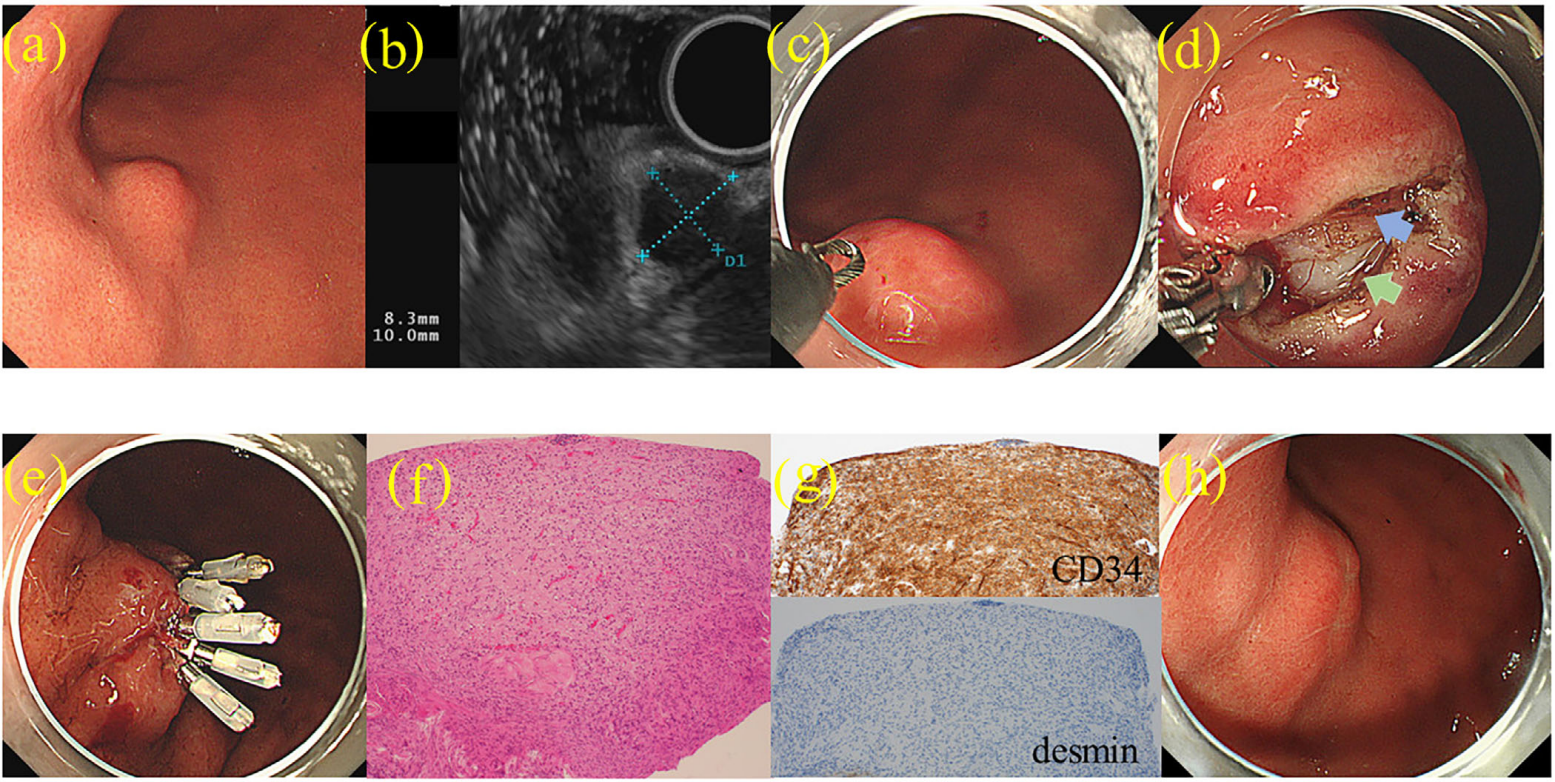
Representative case of mucosal incision‐assisted biopsy: (a) An intraluminal growing type of subepithelial lesion was on the anterior wall at the gastric angle. (b) Endoscopic ultrasonography showed a hypoechoic mass 10 mm in size arising from the fourth layer. (c) In this case, the tip of the electrocautery snare was used for the mucosal incision. (d) The surface of the subepithelial lesion (green arrow) under the pseudocapsule (blue arrow) was exposed. Biopsies were taken under direct visualization of the tumor. (e) The incised mucosa was closed with hemoclips. (f) Pathological examination of the sampled specimen with hematoxylin‐eosin staining. (g) Immunohistochemical staining showed that the lesion was positive for CD34 and negative for desmin. (h) Intraoperative endoscopic findings six months after mucosal incision‐assisted biopsy showed that the incised mucosa had healed.

MIAB was performed as follows: an approximately 10 mm linear mucosal incision was made at the top of the elevation by using a needle‐knife or a tip of the electrocautery snare with the endocut mode, effect 3, and output 40 W. In this incision, regions, where blood flow was assumed to be abundant by preoperative EUS, were avoided. When bleeding occurred, hemostasis was attempted by using the tip of the electrocautery device or the Coagulasper (Olympus) with the soft coagulation mode, effect 7, and output 80 W. The tip of the scope was placed on the incised mucosa to expose the tumor surface as much as possible until the tumor surface was visualized with or without magnifying endoscopy. Subsequently, the tumor tissue was sampled with biopsy forceps under direct visualization several times until the endoscopist was convinced that a sufficient volume of the specimen for histology was collected. Finally, the mucosal incision was closed with hemoclips (HX‐610‐090L; Olympus: Video S1 and Figure 2).

### Postoperative management

The obtained specimens were immediately fixed in 10% formalin and subjected to hematoxylin‐eosin staining followed by immunohistochemical staining with the following antibodies: c‐kit, CD34, S100 protein, and desmin. Patients were observed in the recovery room and were discharged 1 h after the procedure. Oral intake was allowed 2 h after the procedure. Most patients were administered vonoprazan (20 mg/day) for 14 days after MIAB to prevent postprocedure bleeding. All patients were told to come to the outpatient clinic one month after MIAB to be informed of the pathological results.

When MIAB revealed GIST or possible GIST without a definite histological diagnosis, surgery was recommended. In cases of GIST that were finally diagnosed by surgically obtained specimens, the patients were observed according to the GIST guidelines.^22^, ^23^ In cases of non‐GISTs proven by MIAB or surgery, optimal surveillance was recommended according to the histological diagnosis.

### Outcome measures

Regarding the technical outcomes of MIAB, we investigated the success rate of tissue sampling and histopathological diagnosis and compared these results in terms of lesion size (<20 mm vs. ≥20 mm). We also evaluated the minimal number of biopsies to sufficiently obtain the histological diagnosis that was calculated based on the number of biopsies that first led to the diagnosis, the procedure time, and the rate of adverse events. The success rate of tissue sampling and the diagnostic rate were defined as the percentage of lesions in which a sufficient amount of tissue for histological evaluation was obtained and the percentage of lesions in which a definitive diagnosis was obtained, respectively. The procedure time was defined as the duration from the mucosal incision to clip closure.

Furthermore, the postprocedural course of MIAB was investigated. In patients who underwent surgery, the occurrence of the rapid growth of the tumor and the dehiscence of the closure site by clipping on the mucosal incision was investigated, referring to intraoperative endoscopic findings. In addition, the rates of recurrence and metastasis/dissemination during the observational period were assessed. The occurrence of rapid enlargement was defined as a visually apparent increase in the diameter of the lesion from that before MIAB.

### Statistics

Continuous data were expressed as the medians with ranges and were compared using the Mann‒Whitney U test. Categorical variables were expressed as numbers (percentages) and were compared using Fisher's exact test. *p‐*Values <0.05 were employed to indicate statistical significance. A cumulative line graph was drawn by connecting the cumulative definitive diagnostic rate at each number of biopsies and used to analyze the number of biopsies required. All data management and statistical analyses were performed using IBM SPSS version 25 (IBM Corp., Armonk, NY, USA).

## RESULTS

In 61 patients who underwent EUS for precise observation of gastric SELs, 48 patients (79%) with possible intraluminal growth type of GISTs >10 mm were applied to MIAB and enrolled in this study (Figure 1 and Table 1). The median tumor diameter of SELs was 16 mm, with 36 SELs <20 mm and 12 SELs ≥20 mm.

**TABLE 1:** Baseline characteristics of gastric subepithelial lesions in this study.

**Variables**	**Mucosal incision‐assisted biopsy**
(*n* = 48)	
Age (years), median (range)	69 (23–86)
Sex, male, *n* (%)	30 (63)
Lesion location, *n* (%)	
Upper third	18 (38)
Middle third	17 (35)
Lower third	13 (27)
Tumor size in EUS image, *n* (%)	
≧20mm	12 (25)
<20mm	36 (75)
Tumor size in EUS image(mm), median (range) Internal features, *n* (%)	16 (10–35)
Homogenous	16 (33)
Heterogenous	32 (67)
Margin, *n* (%)	
Clear	40 (83)
Unclear	8 (17)
Blood flow, *n* (%)	
Hypervascular	8 (17)
Hypovascular	35 (73)

The clinical outcomes of 48 MIAB cases are summarized in Table 2. Adequate tissues of pathologically evaluable quality and quantity were sampled in 46 cases; the success rate of tissue sampling was 96% (95% confidence interval [CI] 86%–100%). Samples were not successfully collected for the remaining two cases. In one case, lymphocytic collection was observed in tissue sampled by MIAB, but a diagnosis was not confirmed. It was later revealed as schwannoma with a surgical specimen. In the other case, leiomyoma was suspected, but the possibility of the end of GIST could not be denied because of the presence of CD34‐positive cells. Therefore, the definitive pathological diagnosis was obtained in 44 patients (GIST, 23; leiomyoma, nine; schwannoma, one; heterotopic pancreas, 10; malignant lymphoma, one), indicating that the diagnostic rate of MIAB was 92% (95% CI 80%–97%). The modified Flecher classification of GIST cases included four, 15, three, and one cases of very low, low, intermediate, and high risk, respectively. The success rates of tissue sampling and pathological diagnosis were 97% and 94% in lesions less than 20 mm and 92% and 83% in lesions ≥20 mm, respectively.

**TABLE 2:** Technical outcomes and postprocedural course of mucosal incision‐assisted biopsy.

		**Tumor size in EUS image**	
**Variables**	**MIAB**	**<20mm**	**≧20mm**
	(*n* = 48)	(*n* = 36)	(*n* = 12)
Technical outcomes			
Success in tissue sampling, *n* (%)	46 (96)	35 (97)	11 (92)
Success of histopathological diagnosis, *n* (%)	44 (92)	34 (94)	10 (83)
Histopathological diagnosis, *n* (%)			
GIST	23 (48)	15 (42)	8 (67)
Leiomyoma	9 (19)	9 (25)	0 (0)
Schwannoma	1 (2)	0 (0)	1 (8)
Heterotopic pancreas	10 (21)	9 (25)	1 (8)
Malignant lymphoma	1 (2)	1 (3)	0 (0)
Total number of biopsies per case, median (range)	3 (1–9)	3 (1–9)	4 (3–7)
Procedure time (min), median (range)	17 (9–58)	17 (9–58)	22 (12–23)
Intraoperative adverse events, *n* (%)	0 (0)	0 (0)	0 (0)
Postoperative adverse events, *n* (%)			
Bleeding	1 (2)	1 (3)	0 (0)
Perforation	0 (0)	0 (0)	0 (0)
Postprocedural course			
Surgery, *n* (%)	24 (50)	14 (39)	10 (83)
Duration from MIAB to surgery (months), median (range)	2 (1–16)	3 (1–16)	2 (1–8)
Intraoperative endoscopic findings, *n* (%)			
Rapid enlargement	0 (0)	0 (0)	0 (0)
Open ulcer	0 (0)	0 (0)	0 (0)
Observation period (months), median (range)	13 (2–23)	13 (2–23)	17 (2–23)
Recurrence, *n* (%)	0 (0)	0 (0)	0 (0)
Metastasis, *n* (%)	0 (0)	0 (0)	0 (0)

Abbreviations: EUS, endoscopic ultrasound; GIST, gastrointestinal stromal tumor; MIAB, mucosal incision‐assisted biopsy.

**FIGURE 3:**
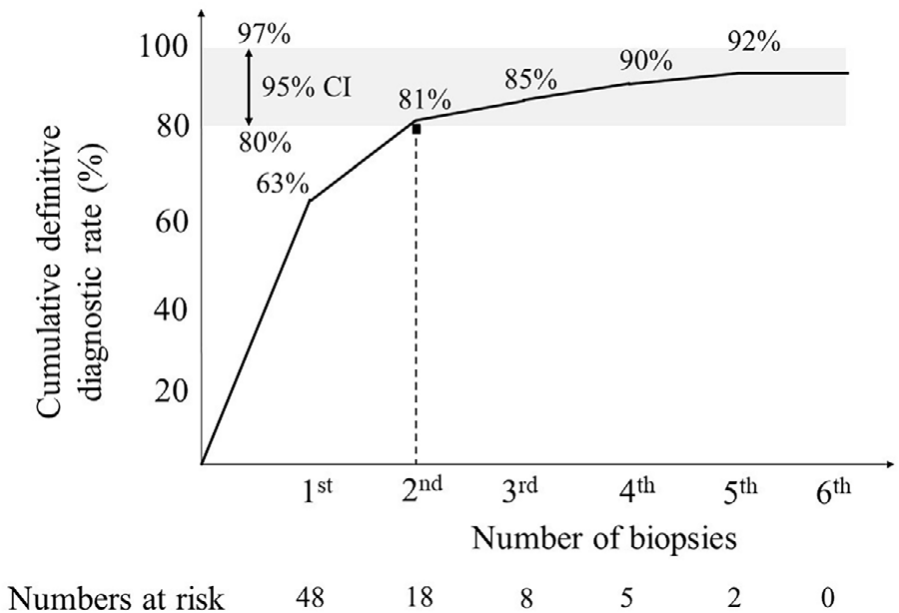
Cumulative line graph of the cumulative definitive diagnostic rate according to the number of biopsies: The cumulative definitive diagnostic rates of each number of times (from first to fifth) were 63%, 81%, 85%, 90%, and 92%, respectively, and no tissue was collected on more than the sixth biopsy in any case. The cumulative line graph shows that the cumulative definitive diagnostic rate exceeded the lower limit of the 95% confidence interval (CI) for the definitive diagnostic rate (80%) at the second biopsy (81%) and thereafter plateaued. In four failed cases of diagnosis, tissue sampling was attempted two, three, four, and five times.

The median number of biopsies per lesion and the median procedure time were three (range 1–9) and 17 (range 9–58) minutes, respectively. A cumulative line graph of the cumulative definitive diagnostic rate was increased at 63%, 81%, 85%, 90%, and 92%, respectively, according to the increase in the number of biopsies, which appeared to generally plateau at the second biopsy (Figure 3).

No intraoperative adverse events occurred, whereas one patient with SEL ≥20 mm experienced haematemesis the day after MIAB. In this case, no bleeding was found on emergency endoscopy, and the patient had no symptoms thereafter without transfusion or hemostatic treatment. Accordingly, the incidence of postoperative bleeding was 2%.

**FIGURE 4:**
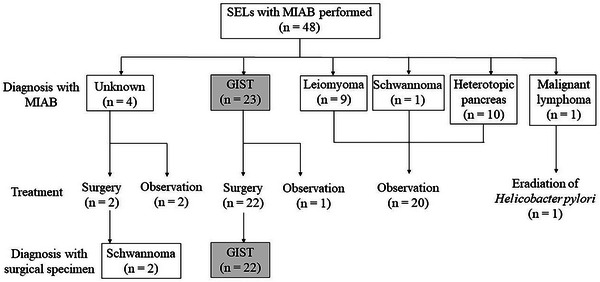
Flowchart of subepithelial lesions sampled by mucosal incision‐assisted biopsy: Of 48 subepithelial lesions (SELs) that underwent mucosal incision‐assisted biopsy (MIAB), 23 lesions were GISTs, of which 22 lesions underwent surgery. Of four SELs that could not be diagnosed with MIAB, two lesions underwent surgery and were revealed to be schwannomas. One case was diagnosed as malignant lymphoma with MIAB, and eradication of *Helicobacter pylori* was performed.

Of 23 patients who were diagnosed with GIST using MIAB, one patient refused to undergo surgery. Of four cases that could not receive a definitive diagnosis by MIAB, two patients underwent surgery, and the other two patients were observed considering a relatively small size and possible leiomyomas from a morphological point of view.^24^ Accordingly, 24 lesions were removed surgically a median of two (range 1–16) months after MIAB. Regarding surgical form, laparoscopic surgery, laparoscopic and endoscopic cooperative surgery, and endoscopic full‐thickness resection under laparoscopic observation were selected in four, 13, and seven cases, respectively. In all 22 surgically removed lesions that were preoperatively diagnosed as GIST by MIAB, the surgical specimens also revealed GIST. The two cases with an indefinite diagnosis at MIAB were both found to be schwannomas. All lesions were removed en bloc with negative surgical margins (Figure 4).

In the 24 surgical cases, lesions had neither rapid enlargement nor open ulcers in intraoperative endoscopic findings. Of the 23 cases diagnosed as other than GIST, one case of malignant lymphoma was treated with the eradication of *Helicobacter pylori*, and six out of the other SELs (27%) were endoscopically observed annually. In a median observation period of 13 (range 2–23) months, including both the surgery group and the observation group, no patient who had undergone MIAB experienced metastasis/dissemination.

## DISCUSSION

Among SELs, GISTs should be correctly differentiated due to their potential malignancy. Although the utility of morphological characteristics and elastography in the diagnosis of these tumors with EUS has been reported, their diagnostic performance is not as good as that of pathological assessment.^24^, ^25^ Therefore, pathological evaluation by tissue sampling is still needed. Recently, the number of small lesions eligible for treatment has increased. Although there is controversy as to whether small SELs should be removed, we consider them eligible for resection to relieve patient anxiety and avoid the risk of follow‐up loss. This concept would be accelerated when performing laparoscopic and endoscopic cooperative surgery or endoscopic full‐thickness resection as the minimally‐invasive endoscopic treatment. Additionally, because MIAB involves an intraluminal incision, its application is theoretically limited to SELs with intraluminal components. For these reasons, we attempted MIAB for gastric SELs ≥10 mm of the intraluminal growth type. In this study, MIAB was feasible in 79% of the eligible lesions, and there was only one case in which the procedure was not indicated due to technical difficulties (this case had both patient factors of poor sedation and high respiratory variability and lesion factors of a small lesion diameter of 10 mm and a location just below the cardia that was difficult to reach with a scope). This suggests that MIAB is an acceptable procedure with many indications.

In this study, the tissue sampling and histological diagnosis rates of MIAB were sufficiently high at 96% and 92%, respectively, and comparable to those previously reported.^11^, ^12^, ^13^, ^14^, ^15^, ^16^, ^17^, ^18^, ^19^ In addition, the diagnostic rate for lesions ≥10 mm and <20 mm was particularly good at 94%, which was higher than that of EUS‐FNA at 67%–71%.^13^, ^15^ Considering the increasing number of small SELs as candidates for less‐invasive surgical removal, the favorable diagnostic rate for lesions <20 mm must be a valuable advantage of MIAB. In addition, surgery is indicated for potentially malignant SEL ≥20mm. Accordingly, we believe that intraluminally growing SELs, especially lesions ≥10 and <20 mm, are the best indications for MIAB. This study showed that definitive diagnosis by MIAB is expected at the second biopsy according to the cumulative line graph of the cumulative definitive diagnostic rate; in fact, the cumulative definitive diagnostic rate at the second biopsy (81%) was above the lower limit of the 95% CI (80%). Based on these results, we suggest two biopsies and believe that multiple biopsies should be avoided considering the risk of adverse events such as bleeding. The reason for this favorable diagnostic rate may be because the lesion is collected under direct visualization, and endoscopists can be convinced that the tissue is sampled on site.

Of the four cases in which definitive diagnoses could not be obtained, two were later surgically resected and pathologically diagnosed as schwannomas; this might indicate the difficulty in tissue sampling and pathological diagnosis of schwannomas. In addition, a larger mucosal incision and wider exposure of the lesions may further improve the tissue sampling rate.

Regarding the adverse events, postoperative bleeding occurred in one case (2%) in this study, which seemed lower than those of previous studies (0%–6.7%).^11^, ^13^, ^14^, ^15^, ^16^, ^17^, ^18^, ^19^ Our lower adverse event rate may be because we prescribed antisecretory agents for 2 weeks after the procedure, but we are not sure whether the use of these agents is truly effective in MIAB due to the small number of cases in this study.

In MIAB, there is a concern that degeneration caused by fever on the surface of tumors by the mucosal incision can cause ulceration and even rapid growth of the tumors. In this study, no cases of rapid growth were observed and the incised mucosa was completely healed in all surgical cases, which implies that there was theoretically no risk of tumor cell seeding into the abdominal cavity during local resection accompanied by transluminal communication. Furthermore, there were no metastatic lesions in the mean follow‐up period of 13 months, both in the surgical and follow‐up cases. This suggests that the abovementioned concern can be ignored regardless of histology including high‐risk cases in modified Flecher classification, although both the duration from MIAB to surgery and the overall observation period in this study were too short to draw definite conclusions. Furthermore, this issue may become relevant when perforation occurs in MIAB.

The strength of this study was to investigate both the diagnostic ability and technical feasibility of MIAB in possible gastric GISTs, including small lesions. However, this study has several limitations. First, the sample size was small, and the data were retrospectively collected at a single institution. In this study, the tissue sampling and histological diagnosis rate of MIAB were slightly lower for lesions ≥20 mm compared to those with lesions ≥10 and <20 mm, but we believe this is due to the small sample size, especially for the latter group. Second, the procedure was performed by only two endoscopists, and the volume of specimens collected by MIAB in each case was left to the discretion of the endoscopist in charge and there were no clear criteria. Third, the oncological effect of MIAB on the postoperative clinical course was not adequately examined. A large, multicentre, prospective study is desired.

In conclusion, MIAB is a reliable tissue sampling method for relatively small, intraluminally growing GISTs with high diagnostic ability, few adverse events, and no negative influence on the postprocedural course. By accumulating further evidence, MIAB is expected to be established and standardized as an alternative to EUS‐FNA in the future.

## CONFLICT OF INTEREST STATEMENT

None.

## Supporting information



Video

## References

[1] Corless CL , Barnett CM , Heinrich MC . Gastrointestinal stromal tumors: Origin and molecular oncology. Nat Rev Cancer 2011; 11: 865–78. 22089421 10.1038/nrc3143

[2] Joesuu H , Hohenberger P , Coreless CL . Gastrointestinal stromal tumour. Lancet 2013; 382: 973–83. 23623056 10.1016/S0140-6736(13)60106-3

[3] Nishida T , Blay JY , Hirota S *et al*. Standard diagnosis, treatment, and follow‐up of gastrointestinal stromal tumors based on guidelines. Gastric Cancer. 2016; 19: 3–14. 26276366 10.1007/s10120-015-0526-8 PMC4688306

[4] Nishida T , Hirota S , Yanagisawa A *et al*. Clinical practice guidelines for gastrointestinal stromal tumor (GIST) in Japan: English version. Int J Clin Oncol 2008; 13: 416–30. 18946752 10.1007/s10147-008-0798-7

[5] Akahoshi K , Sumida Y , Matsui N *et al*. Preoperative diagnosis of gastrointestinal stromal tumor by endoscopic ultrasound‐guided fine needle aspiration. World J Gastroenterol 2007; 13: 2077–82. 17465451 10.3748/wjg.v13.i14.2077 PMC4319128

[6] Moura D , McCarty T , Jirapinyo P *et al*. EUS‐guided fine‐needle biopsy sampling versus FNA in the diagnosis of subepithelial lesions: A large multicenter study. Gastrointest Endosc 2020; 92: 108–19. 32105712 10.1016/j.gie.2020.02.021 PMC7340004

[7] Facciorusso A , Sunny S , Prete V *et al*. Comparison between fine‐needle psy and fine‐needle aspiration for EUS‐guided sampling of subepithelial lesions: A meta‐analysis. Gastrointest Endosc 2020; 91: 14–22. 31374187 10.1016/j.gie.2019.07.018

[8] Yamashita Y , Ashida R , Yamazaki H *et al*. Comparison of 22G fork‐tip and franseen needles and usefulness of contrast‐enhanced endoscopic ultrasound for diagnosis of upper gastrointestinal subepithelial lesions. Diagnostics 2022; 12: 3122. 36553129 10.3390/diagnostics12123122 PMC9776934

[9] Inoue T , Okumura F , Mizushima T *et al*. Assessment of factors affecting the usefulness and diagnostic yield of core biopsy needles with a side hole in endoscopic ultrasound‐guided fine‐needle aspiration. Gut Liver 2016; 10: 51–7. 25963081 10.5009/gnl14249 PMC4694734

[10] Mekky MA , Yamao K , Sawaki A *et al*. Diagnostic utility of EUS‐guided FNA in patients with gastric submucosal tumors. Gastrointest Endosc 2010; 71: 913–9. 20226456 10.1016/j.gie.2009.11.044

[11] Giri S , Afzalpurkar S , Angadi S *et al*. Mucosal incision‐assisted biopsy versus endoscopic ultrasound‐assisted tissue acquisition for subepithelial lesions: A systematic review and meta‐analysis. Clin Endosc 2022; 55: 615–25. 36205045 10.5946/ce.2022.133 PMC9539302

[12] Yamabe A , Irisawa A , Bhutahi M *et al*. Usefulness of endoscopic ultrasound‐guided fine‐needle aspiration with a forward‐viewing and curved linear array echoendoscope for small gastrointestinal subepithelial lesions. Endosc Int Open 2015; 3: E161–4. 26135661 10.1055/s-0034-1391671 PMC4477025

[13] Adachi A , Hirata Y , Kawamura H *et al*. Efficiency of mucosal cutting biopsy for the histological diagnosis of gastric submucosal tumors. Case Rep Gastroenterol 2019; 13: 185–94. 31123445 10.1159/000499442 PMC6514511

[14] Mizukami K , Matsunari O , Ogawa R *et al*. Examine the availability and safety of mucosal cutting biopsy technique for diagnosis of gastric submucosal tumor. Gastroenterol Res Pract 2019; 2: 3121695. 10.1155/2019/3121695 PMC6525918 31191643

[15] Minoda Y , Chinen T , Osoegawa T *et al*. Superiority of mucosal incision‐assisted biopsy over ultrasound‐guided fine needle aspiration biopsy in diagnosing small gastric subepithelial lesions: A propensity score matching analysis. BMC Gastroenterol 2020; 20: 19–28. 31964357 10.1186/s12876-020-1170-2 PMC6975081

[16] Nakano Y , Takao T , Morita Y *et al*. Reasons for diagnostic failure in forty‐five consecutive mucosal cutting biopsy examinations of gastric subepithelial tumors. Clin Endosc 2020; 53: 575–82. 32053861 10.5946/ce.2019.150 PMC7548140

[17] Osoegawa T , Minoda Y , Ihara E *et al*. Mucosal incision‐assisted biopsy versus endoscopic ultrasound‐guided fine‐needle aspiration with a rapid on‐site evaluation for gastric subepithelial lesions: A randomized cross‐over study. Digest Endosc 2019; 31: 413–21. 10.1111/den.13367 30723945

[18] Ikehara H , Li Z , Watari J *et al*. Histological diagnosis of gastric submucosal tumors: A pilot study of endoscopic ultrasonography‐guided fine‐needle aspiration biopsy vs mucosal cutting biopsy. World J Gastrointest Endosc 2015; 7: 1142–9. 26468338 10.4253/wjge.v7.i14.1142 PMC4600180

[19] Nega Y , Dhindsa B , Deliwara S *et al*. Single‐incision needle‐knife biopsy for the diagnosis of GI subepithelial tumors: A systematic review and meta‐analysis. Gastrointest Endosc 2023; 97: 640–5. 36460089 10.1016/j.gie.2022.11.021

[20] Deprez PH , Moons LMG , O'Toole D *et al*. Endoscopic management of subepithelial lesions including neuroendocrine neoplasms: European Society of Gastrointestinal Endoscopy (ESGE) Guideline. Endoscopy 2020; 54: 412–29. 10.1055/a-1751-5742 35180797

[21] Fujimoto K , Fujishiro M , Kato M *et al*. Guidelines for gastroenterological endoscopy in patients undergoing antithrombotic treatment. Dig Endosc 2014; 26: 1–14. 10.1111/den.12183 24215155

[22] Japan Society of Clinical Oncology . Japanese Clinical Practice Guidelines for Gastrointestinal Stromal Tumors (GIST), 4th edn, Tokyo: Kanehara&CO., LTD, 2022.

[23] Nishida T , Blay JY , Hirota S *et al*. The standard diagnosis, treatment, and follow‐up of gastrointestinal stromal tumors based on guidelines. Gastric Cancer 2016; 19: 3–14. 26276366 10.1007/s10120-015-0526-8 PMC4688306

[24] Koizumi E , Goto O , Yoshinaga S *et al*. Circularity is a potential noninvasive diagnostic indicator to differentiate gastric submucosal tumors. Digestion 2022; 103: 287–95. 35405673 10.1159/000523881

[25] Tsuji Y , Kusano C , Gotoda T *et al*. Diagnostic potential of endoscopic ultrasonography‐elastography for gastric submucosal tumors: A pilot study. Dig Endosc 2016; 28: 173–8. 26530730 10.1111/den.12569

